# Supplementary material for: Digital transformation of an academic hospital department: A case study on strategic planning using the balanced scorecard
Source: PLOS Digit Health. 2023 Nov 17;2(11):e0000385. doi: 10.1371/journal.pdig.0000385 (PMC10656018; doi:10.1371/journal.pdig.0000385)
Supplement: S1 Table — (DOCX) [file pdig.0000385.s001.docx]

S1 Table. Implemented digital transformation in our rheumatology department.

| Project | Indicator | Reference |
| --- | --- | --- |
| Regular meetings between clinicians and informaticans | - |  |
| Workshops for medical doctors in data science and Python coding | Publications, digital care pathways | - |
| Yearly international conference on Digital Rheumatology, creation of the Digital Rheumatology Network | Conference participants, LinkedIn followers | www.digitalrheumatology.org |
| Prediction the disease activity of rheumatoid arthritis at next visit by deep learning | Digital care pathway for rheumatoid arthritis | (*1*) |
| Clustering / phenotyping of rheumatoid arthritis patients | Treatment efficacy, Digital care pathway for rheumatoid arthritis | (*2*) |
| Clustering in fibromyalgia and chronic pain patients and response to a multimodal treatment program | Digital care pathway for chronic pain patients | (*3*) |
| Automated image recognition and report creation for DEXA | Digital care pathway for osteoporosis | (*4*) |
| DETECTRA, a digital biomarker for joint swelling | Digital care pathway for rheumatoid arthritis | (*5*) |
| Automated image recognition and grading of hand osteoarthritis | Automatization, Digital care pathway for osteoarthritis, quality management | Manuscript in preparation |
| Tight-safety control via telemonitoring for difficult to treat patients with rheumatoid arthritis | Efficacy (DAS28), Digital Care Pathway in rheumatoid arthritis | Manuscript in preparation |
| Development and user experience of remote safety monitoring during biologic treatment | Safety | - |
| Quality control by a national registry (SCQM) with an app for PROs | Treatment efficacy, safety | - |
| Physical interdisciplinary exchange by the ‘Common Ground Meeting on Immune-Mediated Diseases’ | Participants | [www.common-ground-meeting.org](http://www.common-ground-meeting.org) |
| Prediction of hospital stay and case mix | Duration of hospital stay, DRG | - |

REFERENCE

1. M. Kalweit *et al.*, Personalized prediction of disease activity in patients with rheumatoid arthritis using an adaptive deep neural network. *PLoS One* **16**, e0252289 (2021).

2. p. c. M. style="margin-top:12.0pt *et al.* (PLOS Computational Biology (conditionally accepted), 2023).

3. T. Hügle, T. Pretat, M. Suter, P. Ming-Azevedo. (EULAR Abstract  P0198, 2023).

4. E. Shevroja, O. Lamy, E. Gonzalez Rodriguez, D. Hans, [Towards an individualized bone health report focused on the patient - Bone parameters and treatment options]. *Rev Med Suisse* **17**, 774-779 (2021).

5. T. Hügle *et al.*, Dorsal Finger Fold Recognition by Convolutional Neural Networks for the Detection and Monitoring of Joint Swelling in Patients with Rheumatoid Arthritis. *Digit Biomark* **6**, 31-35 (2022).
