# Supplementary material for: Digital transformation of an academic hospital department: A case study on strategic planning using the balanced scorecard
Source: PLOS Digit Health. 2023 Nov 17;2(11):e0000385. doi: 10.1371/journal.pdig.0000385 (PMC10656018; doi:10.1371/journal.pdig.0000385)
Supplement: Attachment — Submitted filename: Point to point discussion BSC.docx [file pdig.0000385.s002.docx]

**First all we would like to express our gratitude to the editor and the reviewer for their work spend on this article.**

**The manuscript was initially written as opinion rather than a research paper, thus the methodology has been sparse. We therefore rewrote substantial parts of the manuscript and changed it to a more scientific case-study. The figures have also been revised. The positive and stimulating points from the reviewer and editor were very much appreciated and helped to improve the article.**

**1. Please provide separate figure files in .tif or .eps format only and remove any figures embedded in your manuscript file. Please also ensure that all files are under our size limit of 10MB.**

OK

**2. We noticed that you used "unpublished" in the manuscript. We do not allow these references, as the PLOS data access policy requires that all data be either published with the manuscript or made available in a publicly accessible database. Please amend the supplementary material to include the referenced data or remove the references.**

OK

**3. We have noticed that you have uploaded Supporting Information files, but you have not included a list of legends. Please add a full list of legends for your Supporting Information files after the references list.**
OK

Additional Editor Comments (if provided):

**Although the reviewers found the manuscript promising, it is not quite as organized as we need it to to both be easily digestible for the reader and to conform with PLOS requirements. Specifically, I would discourage the use of the numbered list in the abstract. Also, per PLOS authors' guidelines, "While the Abstract is conceptually divided into three sections (Background, Methodology/Principal Findings, and Conclusions/Significance), do not apply these distinct headings to the Abstract within the article file."**

**Similarly, it might not be wise to make the discussion long and discuss the four issues that have separate headlines (some of this might be more for the discussion).**

Thank you, we performed substantial edits in this regard.

**The end of the introduction lacks a clear goal/objective.**

OK this has been added.

**For the methods & materials, consider using more references if you don't want to spell out your methods (e.g., software used, prior categorization etc) too much.**

We have included more references in the methods.

**A part of the results likewise contains elements that normally appear in the
discussion, e.g., that you plan to use ChatGPT next.**

**The entire results section might benefit from a reorganization, so that the reader can follow more easily. Results don't usually contain bullet lists; consider a table for it.**

OK. We reorganized the results section.

**The discussion should start with a high-level summary of your results. Remember that parts of the introduction and results should be moved to the discussion, as appropriate.**

OK. We changed this accordingly.

**My main criticism is that at the end of the discussion, what appears to be the conclusion is far too general; try to put in the implications here - should other implement BSC approaches, or is it too early (or would you recommend against it)? What should be kept in mind, and what needs to be researched further? We look forward to your revision.**

Reviewers' comments:

Reviewer #1:
**First, the introduction has to be re-written with a clear definition of the horizontal digital transformation.**

Thank you for this comment we restructured substantial parts of the introduction. It should be clearer now. In fact, also responding to other comments, we reshaped the scope of this article on horizontal and vertical digital transformation (with the focus on the BSC).

**The Balanced scorecard (BSC) has to be defined more clearly in the introduction.**

OK, we explained the BSC in more detail.

**The aim does not mention the utilization of BSC. It is not clear how the new opportunities were connected with BSC or the methodology.**

Agree. We included the utilization of BSC in the aims and explain how opportunities are connected with BSC.

**The importance and knowledge gap of this manuscript are not presented. Why did the authors wrote this manuscript? what are the practical implications?**

This is correct. We included my motivation and the gap of knowledge why we wrote this manuscript.

**Regarding the methodology, it lacks a description of the time frame of implementation**.

This point is well taken. We included the time frame of 2 years in the manuscript.

**Also it is not clear how BSC is connected to it. Usually, the KPIs are designed by a panel or committee. The face and content validity, or the measurability and feasibility are assessed before implementation. Also, the figure used is not clear. the source of each KPI is not clear. The internal process perspective and knowledge and information perspective are mixed together.**
We agree with this point. The main focus of this paper is to show a method for the strategic planning. Not all of our interventions have been implemented. Thus, not all KPI have been validated at this point, but most of them were identified and assessed for measurability and feasibility in the working groups. We clarify this in the article now.

Internal process perspective was restructured in operations management, innovation and development, customer service and regulatory and compliance.

**Other minor comments: the language have to be revised. Examples: 1- suported instead of supported in the abstract, 2- ect instead of etc 3- undadressed problem instead of unaddressed.** 
Thank you. We have edited the manuscript.

**Also the authors referred to the four perspectives in the methodology as four key performance indicators. This has to be corrected. To understand the design of BSC further we recommend to have a look on the BSC perspectives, dimensions, and KPIs (**[**https://doi.org/10.1186/s12913-022-07863-0**](https://doi.org/10.1186/s12913-022-07863-0)**).**
Thank you, this reference was helpful. We explain in the manuscript our choice of KPIs.

**Reviewer #2: How managing digital transformation implementation in healthcare is a timely challenge. Moreover, I found a promising case study described in this manuscript. However, I was overwhelmed by the rich flow of information and the manuscript in this release appears out-of-focus. I set below a list of recommendations that would assist the authors while revising their study.**

Thank you, indeed this is helpful. We streamlined the information provided in the article.

**1. What is the real aim of the study? The abstract does not mention it.**

The abstract has been restructured and includes the aim now.

**In the introduction section you mention two aims, firstly “In this article, we aim to show how digital solutions respond to those clinical and administrative needs and what is needed to implement them in clinical practice”; secondly “This article aims to present a scalable and understandable matrix to instruct and foster horizontal digital transformation in hospitals from a medical side”. And the following sections describe much more than these two aims. Probably, your case study could provide data for more than one article, but each article must have a proper research question and the empirics will provide an answer to it. For example, in this article, you describe 1. causes of horizontal integration of digital solutions, 2. Solutions that would implement, 3. Solutions implemented, 4. Results of the implementation. In my opinion, you have to choose your focus and describe properly one of these points. The others could be at most briefly mentioned. You could revise entirely the manuscript according to the novel angle you choose to describe.**

Thank you for this important point. We agree that the paper was not focused enough. However, we don´t agree on points 1. and 4. We don´t describe the cause of horizontal integration of digital solutions and we don´t show results of implemented solutions (meaning we don´t measure them yet).

The actual focus of this work is the BSC as a tool to implement and estimate (and later to measure) the impact digital solutions in a medical department. We restructured the whole article in order to make this clearer.

**2. Why do you consider the balanced scorecard a proper framework to refer to your story? The Balanced Scorecard is a performance management system that aims to help the execution of strategy at the organizational level. Moreover, this system presents four perspectives and the key performance indicators identified in each of these perspectives present a cause-effect link. Thus, your use at the project level, with other perspectives and without a clear identification of leading and lagging indicators should be explained. The balanced scorecard is a powerful management system, but it is not a roadmap.**

Thank you for this clear suggestion that we fully adapt in the new version of the paper.

**It requires a careful design and implementation process and the creation of strategy maps. I suggest reading papers about balanced scorecard implementation and its use in organizational contexts. Hope this published paper could be of any help.**

**Tawse A., Tabesh P. (2023). Thirty years with the balanced scorecard: What we have learned. Business Horizons, 66(1), 123-132.
Ming-Chin Y., Yu-Chi T. (2006), Using Path Analysis to Examine Causal Relationships Among Balanced Scorecard Performance Indicators for General Hospitals. The Case of a Public Hospital System in Taiwan. Health Care Management Review, 31(4), 280-288.
Epstein M., Manzoni J-F (1997), The balanced scorecard and tableau de bord: Translating strategy into action. Management Accounting, 79(2), 28-36.**

We read the paper and cite them partly, that was very helpful.

**3. The methodological section is completely skipped. The methodological rigour in collecting and analysing data is very important. You have a good entrance on the case study and maybe you managed the entire transformation process. Hence, you could benefit from a lot of details: documents, notes of informal meetings, and interviews. If you actively participate in the implementation process, you could approach the action/interventionist research method. It requires adequate theoretical preparation to be distinguished from consultancy, but it could give you a lot of satisfaction. You could analyse these studies.**
Baard, V. (2010), "A critical review of interventionist research", Qualitative Research in Accounting & Management, Vol. 7 No. 1, pp. 13-45. <https://doi.org/10.1108/11766091011034262>
Lukka K., Wouters M. (2022), Towards interventionist research with theoretical ambition, Management Accounting Research, 55, <https://doi.org/10.1016/j.mar.2022.100783>

This is correct. We now have integrated this in the methodology.

**Reviewer #3: This paper instructs and foster horizontal digital transformation in a Swiss academic rheumatology department, using a Balanced Scorecard approach.
The subject is very interesting and relevant.
The paper is well-written and structured.
The approach of the balanced scorecard was adequate. The perspectives were well-adjusted to the case and aligned with the mission, vision, and values.
I recommend removing abbreviations from the abstract (eg ERM).
Despite liking the title, I would consider replacing it and including the “balanced scorecard” in it (for example** **Horizontal Digitalization in Hospitals using Balanced Scorecard: a Roadmap towards Connected Care)**

Thank you. We have changed the title including the term case study. After reflection we left the term balanced scorecard out of the title but included it in the keywords, and of course in the abstract.  **Reviewer #4: your research objective is to present the Case of your institution's works in digital transformation. Why should use the Balanced Scorecard to do this research? what is the relationship between the method results and the routine?where is the Data analysisi from BS method? the paper should be based on the medical demand to put forward the routine and describe the details of that.another the paper's format should be improved.**

Thank you very much. We now explain better why we used the Balanced Scorecard as tool for the strategic planning of the integration of digitalization in our department. We did not perform analyses on causal linkage of variables or KPIs in this project on strategic planning. We made the medial demand clearer.

**Reviewer #5: Dear Author,
Thank you for your contribution.
Although the paper focuses on a hot topic, there are several issues to improve.
The main limitation of the paper is the methodology, how did you collect data? how did you build the balanced scorecard? how did you identify the indicators?**

We agree with this important point that has also been raised by the other reviewers. We have now included the full methodology of this case study including literature reviews, semi-structured reviews, SOAR analysis. We also provide more information about who (and based on which data) the Balanced Scorecared has been adapted.

**Moreover there are other few comments:
- the abstract is quite longer than the limit fixed by the journal and the aim is not clear;
- the first part of the findings (until the implementation section) is not clear because of the methodology, it is unclear how you built the results by using the BSC.
Finally, please, check the english language.**

Thank you very much In the new version of the manuscript we address all those issues. **--------------------

Reviewer #1: Yes: Faten Amer

Reviewer #2: No

Reviewer #3: No

Reviewer #4: Yes: Xinli Zhang

Reviewer #5: No

--------------------


[NOTE: If reviewer comments were submitted as an attachment file, they will be attached to this email and accessible via the submission site. Please log into your account, locate the manuscript record, and check for the action link "View Attachments". If this link does not appear, there are no attachment files.]**
