# Supplementary material for: Digital transformation of an academic hospital department: A case study on strategic planning using the balanced scorecard
Source: PLOS Digit Health. 2023 Nov 17;2(11):e0000385. doi: 10.1371/journal.pdig.0000385 (PMC10656018; doi:10.1371/journal.pdig.0000385)
Supplement: Attachment — Submitted filename: Point to point discussion BSC_1_9_23.docx [file pdig.0000385.s003.docx]

Response to reviewers

Thank you, the double abbreviations (e.g. for PROs, EMR or KPI) have been corrected.
